# Exo70 intracellular redistribution after repeated mild traumatic brain injury

**DOI:** 10.1186/s40659-021-00329-3

**Published:** 2021-02-16

**Authors:** Matías Lira, Pedro Zamorano, Waldo Cerpa

**Affiliations:** 1 Departamento de Biología Celular y Molecular, Facultad de Ciencias Biológicas, Pontificia Universidad Católica de Chile, Av. Libertador Bernardo O´Higgins 340, Santiago, Chile; 2 Centro de Excelencia en Biomedicina de Magallanes (CEBIMA), Universidad de Magallanes, Punta Arenas, Chile; 3 Departamento Biomédico, Universidad de Antofagasta, Antofagasta, Chile; 4 Instituto Antofagasta, Universidad de Antofagasta, Antofagasta, Chile

**Keywords:** Hippocampus, Exocyst, Exo70, Compartmentalization, Redistribution, Traumatic brain injury

## Abstract

**Background:**

Exo70 is a subunit of the greater exocyst complex, a collection of proteins that oversees cellular membrane addition and polarized exocytosis by acting as a tethering intermediate between the plasma membrane and newly synthesized secretory vesicles. Although Exo70 function has been implicated in several developmental events including cytokinesis and the establishment of cell polarity, its role in neuropathologies is poorly understood. On the other hand, traumatic brain injury is the result of mechanical external force including contusion, fast acceleration, and expansive waves that produce temporal or permanent cognitive damage and triggers physical and psychosocial alterations including headache, memory problems, attention deficits, difficulty thinking, mood swings, and frustration. Traumatic brain injury is a critical health problem on a global scale, constituting a major cause of deaths and disability among young adults. Trauma-related cellular damage includes redistribution of N-methyl-D-aspartate receptors outside of the synaptic compartment triggering detrimental effects to neurons. The exocyst has been related to glutamate receptor constitutive trafficking/delivery towards synapse as well. This work examines whether the exocyst complex subunit Exo70 participates in traumatic brain injury and if it is redistributed among subcellular compartments

**Results:**

Our analysis shows that Exo70 expression is not altered upon injury induction. By using subcellular fractionation, we determined that Exo70 is redistributed from microsomes fraction into the synaptic compartment after brain trauma. In the synaptic compartment, we also show that the exocyst complex assembly and its interaction with GluN2B are increased. Finally, we show that the Exo70 pool that is redistributed comes from the plasma membrane.

**Conclusions:**

The present findings position Exo70 in the group of proteins that could modulate GluN2B synaptic availability in acute neuropathology like a traumatic brain injury. By acting as a nucleator factor, Exo70 is capable of redirecting the ensembled complex into the synapse. We suggest that this redistribution is part of a compensatory mechanism by which Exo70 is able to maintain GluN2B partially on synapses. Hence, reducing the detrimental effects associated with TBI pathophysiology.

**Supplementary Information:**

The online version contains supplementary material available at 10.1186/s40659-021-00329-3.

## Background

The exocyst is an evolutionarily conserved protein complex that is composed of eight subunits: Sec3, Sec5, Sec6, Sec8, Sec10, Sec15, Exo70 and Exo84 [1–3] first identified in yeast and then characterized in superior eukaryotic cells. It is a complex that oversees secretory vesicle tethering at the plasma membrane before SNARE-mediated vesicle fusion during exocytosis [4–7]. To date, the exocyst complex has been found to participate in several cellular processes, including cell division [8], polarized exocytosis and membrane growth [1, 9, 10], glutamate receptor trafficking [11–13], and membrane targeting of the glucose transporter GLUT4 [14] among others. Additionally, exocyst assembly has been proven to be a key event in the activity of the complex. The more assembled complex, the more activity on exocytosis [15, 16].

Exo70 is one of the most studied exocyst subunits. Exo70 is localized at the plasma membrane and can directly interact with regions containing phosphoinositol-4,5-bisphosphate (PI(4,5)P_2_) through a phospholipid-binding motif located in its C-terminus [17, 18]. In doing so, it mediates the exocyst targeting to the plasma membrane [19]. Exo70 has been shown to participate in neurite development and synapse formation/stabilization [20, 21]. Its intracellular localization is important to carry on its tethering and exocytosis functions [14, 20, 22, 23] and particularly its plasma membrane localization promotes the activity of the complex [14, 24, 25].

The knowledge of exocyst’s involvement in brain pathologies is very limited. At the moment there are only case reports indicating that Sec15 downregulation is involved in neurodevelopmental abnormalities affecting ultimately intellectual ability, language delay, and speech capacity [26, 27]. Outside of those reports, none has studied exocyst’s involvement in neurodegenerative diseases or particularly in acute neuropathologies like traumatic brain injury.

Traumatic brain injury (TBI) is a neurological injury that is a critical health problem worldwide. TBI leads to high medical costs [28] and it’s recognized as a major cause of permanent disability and death among young adults [29]. It is caused by mechanical external forces applied to the brain causing primary cellular damage (i.e. axonal injury, tissue deformation, and plasma membrane disruption) [30] which leads to secondary damage like excitotoxicity, oxidative stress, neuroinflammation, and ultimately, cell death [31]. Most common causes of TBI include car accidents, contact sports, falls, among others and its most frequent form is mild traumatic brain injury (mTBI) depicting 80–90% of all TBI cases [30, 32, 33]. One of the cellular and molecular mechanisms that have been studied in TBI pathophysiology is the signaling associated with the N-methyl-D-aspartate receptor (NMDAR) [31]. NMDAR can be found both in synaptic and extrasynaptic compartment producing signaling that has to be strictly balanced to get a functional neuron [34]. When synaptic/extrasynaptic signaling balance is disrupted towards extrasynaptic signaling, detrimental outputs are triggered and induces neuron malfunctioning [35]. This disbalance is a relevant aspect of TBI pathophysiology development [31]. Many studies have related trafficking and exocytosis impairments in TBI [36–40], raising the possibility that proteins related to those processes might be important to injury development. Specifically, NMDAR is redistributed outside of the synapse upon TBI induction [41] and thus receptor availability at synapse should be modulated by endocytosis, trafficking, and/or exocytosis.

Taken together, the exocyst complex through Exo70 might be relevant in mTBI pathophysiology development participating in trafficking or exocytosis processes. In the current study, we have examined whether Exo70 function is altered upon mTBI induction. By assessing Exo70 intracellular localization and exocyst assembly we determined that Exo70 is redistributed into synapse after mTBI where the complex assembly is increased. Also, we determined that Exo70-NMDAR interaction is increased as well.

## Results

### Evaluation of Exo70 expression

**Fig. 1:**
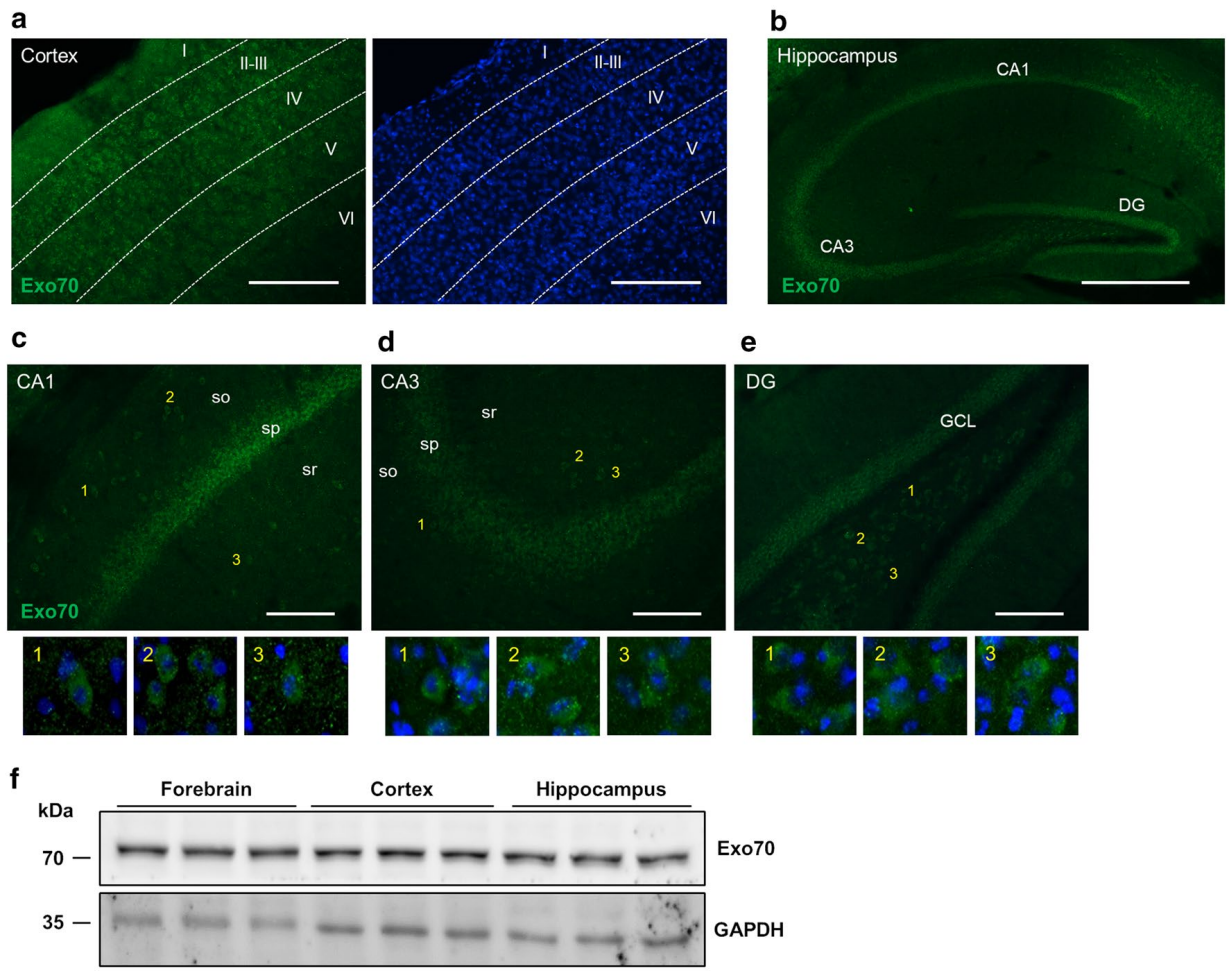
Evaluation of Exo70 expression in the brain, cortex, and hippocampus. Two-month-old male mice were perfused and 30 µm thick brain slices were prepared. **a**, **b** Representative images of slices obtained between 1.8 and 2 mm caudal from bregma. Slices were stained with an Exo70 specific polyclonal antibody and Hoechst to label the nucleus. Cortical layers and hippocampal zones are depicted in the images. Scale bars: cortex 300 µm, hippocampus 400 µm. **c**, **d**, **e** Hippocampal zones CA1, CA3, and DG are shown. so: Stratum oriens; sp: Stratum pyramidale; sr: Stratum radiatum. Scale bars: 100 µm. **f** Western blot analysis of brain, cortex, and hippocampus protein extracts. Samples were resolved in 10% SDS-PAGE. 30 µg of protein samples were loaded in each well. n = 3 mice per group

Exo70 expression has been reported in rat and mouse forebrain [21, 42]. Nevertheless, a more profound description in structures like cortex or hippocampus is yet to be described. We analyzed cortex and hippocampus slices of 2 months old C57BL/6J mice for Exo70 expression and localization. By immunohistochemistry, Exo70 is observed in mostly all cortical layers. Most of the signal comes from II to III layers and the immunoreactivity is reduced in inner layers V and VI (Fig. 1a). Hippocampus also shows Exo70 expression throughout all hippocampal zones (Fig. 1b). Exo70 immunoreactivity is predominantly observed in CA1 and CA3 *stratum pyramidale*, although some Exo70-positive cells in *stratum oriens* and *stratum radiatum* are shown on the lower panels (Fig. 1c, d). Finally, dentate gyrus Exo70 expression is predominantly observed in the granule cell layer (Fig. 1e) and immunoreactivity is detected in hilus-located cells (Fig. 1e, lower panels). Next, we obtained protein extracts from the forebrain, cortex, and hippocampus from 2-month-old mice, and samples were analyzed by western blot. The analysis shows that Exo70 is expressed on the forebrain, cortex, and hippocampus (Fig. 1f) supporting immunohistochemistry results.

### Exo70 compartmentalization following mild traumatic brain injury

**Fig. 2:**
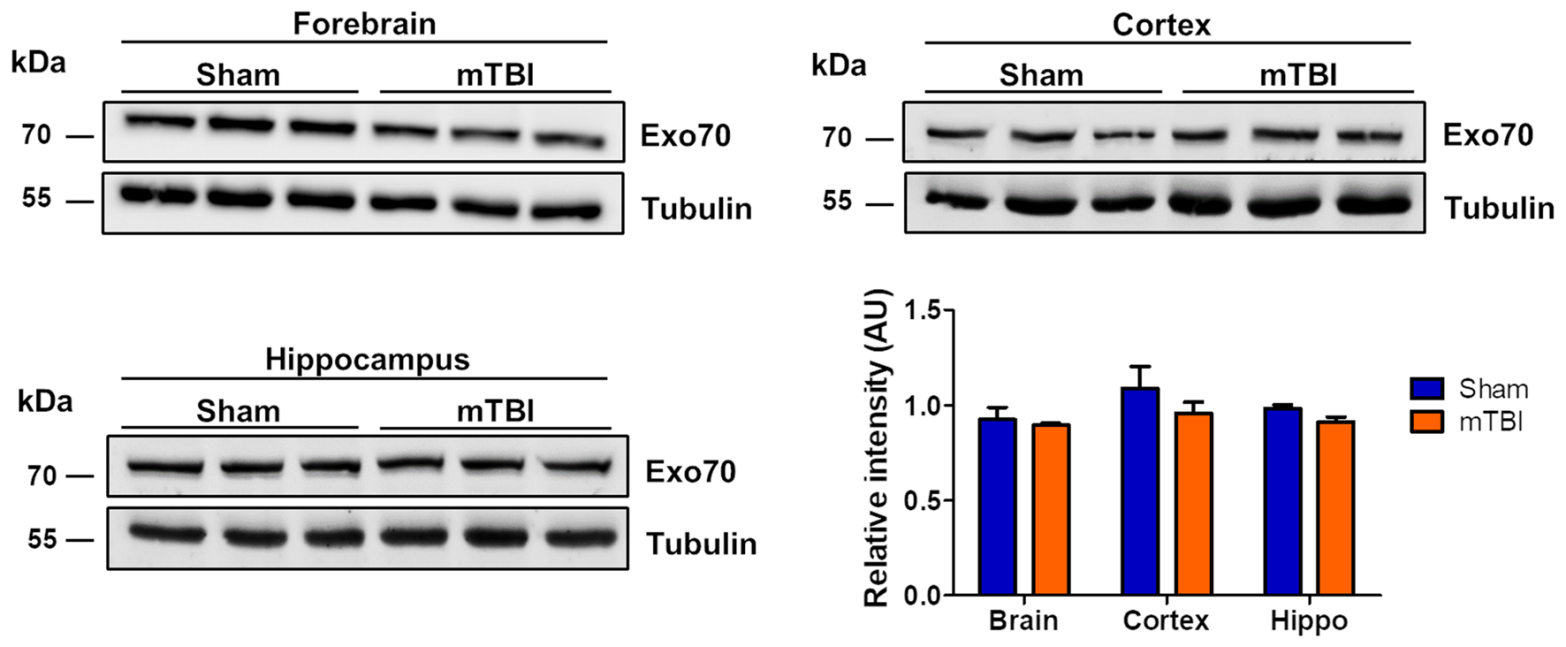
Exo70 protein levels are not altered after mTBI induction. Two-month-old male mice were subjected to mTBI and protein extracts were prepared from the forebrain, cortex, and hippocampus. Protein samples were resolved in 10% SDS-PAGE and transferred to PVDF membrane. Western blot analysis was carried out using Exo70 and Tubulin antibodies. The graph shows the Exo70 densitometric analysis normalized with the Tubulin signal. Values represent means ± SEM, n = 3 mice per experimental group. Statistical differences were determined by an npaired t-test comparing Sham and mTBI. Brain *p* = 0.18, Cortex *p* = 0.29, Hippocampus *p* = 0.37

The exocyst is an octameric complex mainly related to trafficking and exocytosis. While the exocyst has been related to cancers or cell invasion, its role in brain diseases is poorly understood. One case particularly has shown that the downregulation of Sec15 presents epileptic and minor dysmorphic features alongside reduced speech capacity resembling autism [26]. Other case points out to intellectual disability, language delay, facial asymmetry, and ear malformation when Sec15 gene function abnormally [27]. Both cases resemble neurodevelopmental disorders, but no studies have been conducted to evaluate the contribution of exocyst complex in neurodegenerative diseases or particularly in acute neuropathologies like mTBI. To induce mTBI, we used a modified Maryland’s weight drop model used for rats [43] to fit mouse anatomy. This model uses a repeated scheme (see methods) in which brain damage is achieved [44]. Our model induces diffuse damage in the cortex that eventually reaches the hippocampus provoking impaired synaptic transmission and cognitive decline (data not shown). Initially, we sought to determine if mTBI alters Exo70 expression in the forebrain, cortex, and hippocampus. Protein samples were obtained and analyzed by Western blot. The analysis showed that Exo70 protein level was not altered when mice were subjected to TBI in all anatomical regions (Fig. 2).

**Fig. 3:**
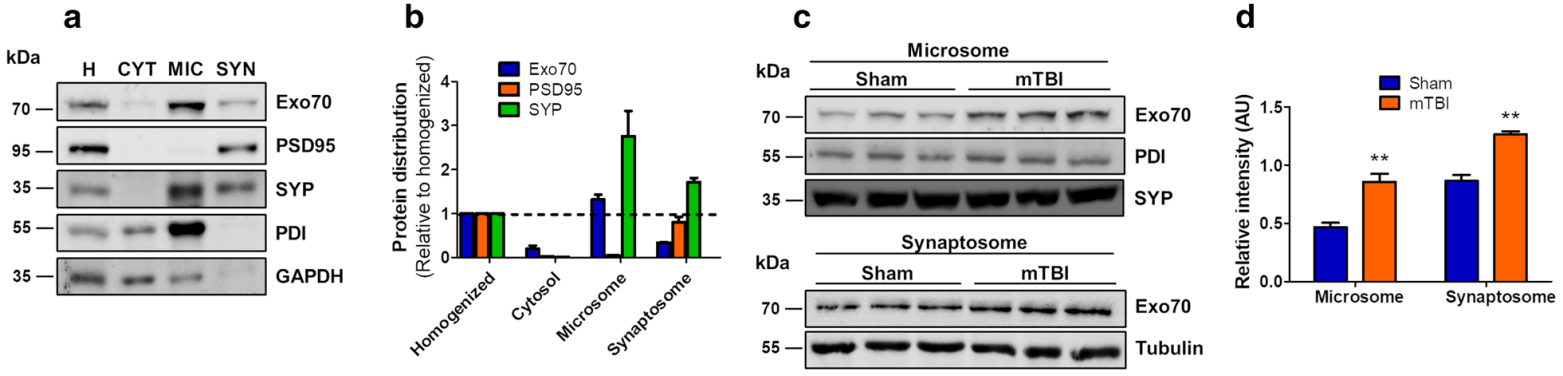
Exo70 localization is increased in forebrain synapses upon mTBI induction. **a** Forebrains from two-month-old male mice were obtained and subcellular fractionation was carried out. Homogenized (H), cytosol (CYT), microsome (MIC), and synaptosome (SYN) fractions were obtained. 20 µg of protein samples were resolved in a 10% SDS-PAGE and transferred to PVDF membranes. Membranes were incubated with the respective antibodies shown in the figure. Membranes were stripped and tested again with the indicated antibodies. **b** Proteins distribution were analyzed with densitometric analysis by comparing signal intensity from each fraction with homogenized. Mean values ± SEM are shown. **c** Two-month-old male mice were subjected to mTBI and microsome and synaptosome fractions were obtained. 30 µg of protein samples were analyzed. PDI/Tubulin was used as loading controls. **d** The graph shows the Exo70 densitometric analysis normalized with loading controls. Values represent means ± SEM, n = 3 mice per experimental group. Statistical differences were determined by an unpaired t-test comparing Sham and mTBI. **p < 0.01

Exo70, as part of the exocyst complex, has shown to have several functions. Most of them related to membrane trafficking and exocytosis [45], particularly localized near the plasma membrane [24, 25]. Indeed, the correct function of Exo70 strictly depends on its subcellular localization [14, 20, 25, 46]. To study this, we used an old biochemical approach to assess if mTBI changes the subcellular patterning in tissue. Forebrain subcellular fractionation was carried out using a well-established protocol [21, 47] to obtain cytosol, microsome, and P2 crude membrane fraction. P2 is then further fractionated to obtain synaptosomes. Samples were processed and analyzed by western blot (Fig. 3a). As expected, the major postsynaptic scaffolding protein PSD95 was mainly distributed in synaptosomes (Fig. 3b). Synaptophysin, a synaptic vesicle protein, was highly distributed in microsomes and synaptosomes (Fig. 3b). Protein disulfide-isomerase (PDI) is an endoplasmic reticulum-resident protein and therefore is distributed in microsome fractions. GAPDH, a cytosolic protein, was distributed in cytosolic fraction (Fig. 3b). Hence, the distribution of those proteins validates the biochemical preparations. Exo70 was present in cytosol, microsome, and synaptosome fractions from the forebrain and was highly distributed in microsomal fraction (Fig. 3a, b). Then, mice were subjected to mTBI, and forebrains were fractionated to obtain microsomal and synaptosomal fractions (Fig. 3c). In these fractions, Exo70 localization was increased on mTBI mice compared to Sham mice (Microsome: Sham 0.46 ± 0.03, mTBI 0.85 ± 0.06; Synaptosome: Sham 0.86 ± 0.05, mTBI 1.26 ± 0.02) (Fig. 3d) suggesting that Exo70 may increase its functionality upon mTBI induction.

**Fig. 4:**
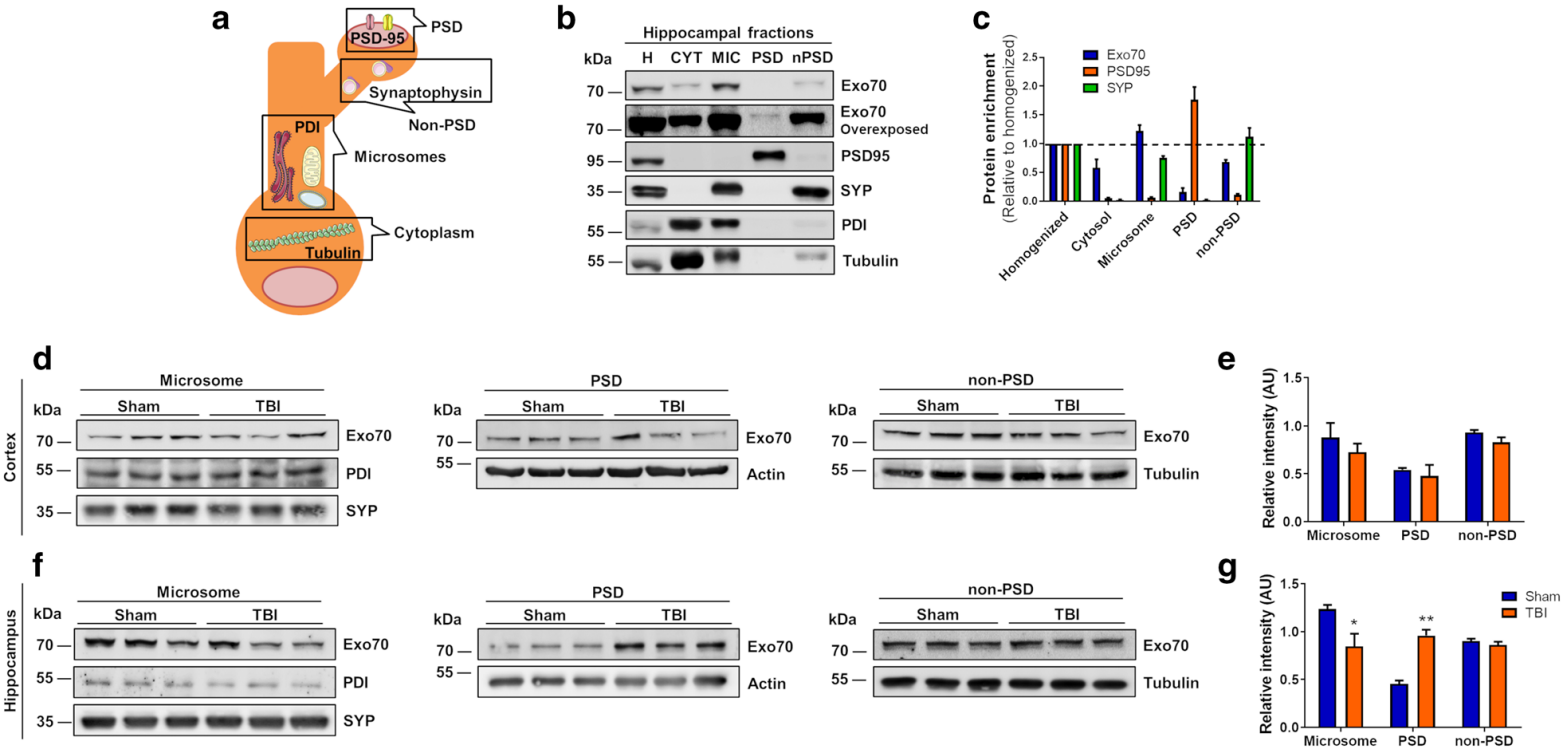
Exo70 is redistributed into PSD in the hippocampus of mTBI mice. **a** Schematic representation of subcellular fractionation proteins. **b** Example of subcellular fractionation. Hippocampus from two-month-old male mice was fractionated and microsome, PSD, and nonPSD fractions were obtained. 20 µg of protein samples were resolved in a 10% SDS-PAGE and transferred to PVDF membranes. Membranes were incubated with the respective antibodies shown in the figure. Membranes were stripped and tested again with the indicated antibodies. **c** Proteins distribution were analyzed with densitometric analysis by comparing signal intensity from each fraction with homogenized. Mean values ± SEM are shown. **d** Cortex and Hippocampus (**f**) from Sham and mTBI mice were fractionated and analyzed by western blot using Exo70, PDI, Actin, and Tubulin antibodies. PDI/Actin/Tubulin was used as loading controls. 30 µg of protein samples were used. **e**, **g** The graph shows the Exo70 densitometric analysis normalized with loading controls. Values represent means ± SEM, n = 3 mice per experimental group. Statistical differences were determined by an unpaired t-test comparing Sham and mTBI. *p < 0.05, **p < 0.01

Because the damage induced by mTBI can reach the hippocampus passing through the cortex, we sought to determine if Exo70 subcellular distribution would behave similarly between both cortex and hippocampus. To test this, we carried out subcellular fractionation using the same protocol for the forebrain and analyzed it again by western blot. An example of hippocampi subcellular fractions are shown in Fig. 4b. In these experiments, synaptosome fraction was treated with Triton X-100 and further fractionated to obtain PSD and nonPSD fractions (Fig. 4a). PSD95 showed that is highly distributed in PSD and excluded from nonPSD fraction (Fig. 4c). Contrary to this, Synaptophysin was highly distributed in nonPSD and excluded from PSD fraction (Fig. 4c) indicating that synaptosome fractionation worked as expected. Exo70 was again highly distributed in microsomes (Fig. 4b). However, it was present in PSD and nonPSD fractions, although most of the synaptosomal Exo70 was present in nonPSD. Next, we performed subcellular fractionation of cortex and hippocampus obtained from Sham and mTBI mice. Exo70 distribution was analyzed in microsome, PSD, and nonPSD fractions by western blot (Fig. 4d and f). No Exo70 distribution changes were observed on the cortex (Fig. 4e) while Exo70 distribution in the hippocampus was altered upon mTBI induction. Specifically, Exo70 localization was reduced on microsome and increased in PSD fraction (Microsome: Sham 1.23 ± 0.04, mTBI 0.85 ± 0.13; PSD: Sham 0.45 ± 0.03, mTBI 0.96 ± 0.06; nonPSD: Sham 0.90 ± 0.02, mTBI 0.86 ± 0.03) (Fig. 4g), suggesting that Exo70 is redistributed from microsome to PSD following mTBI.

**Fig. 5:**
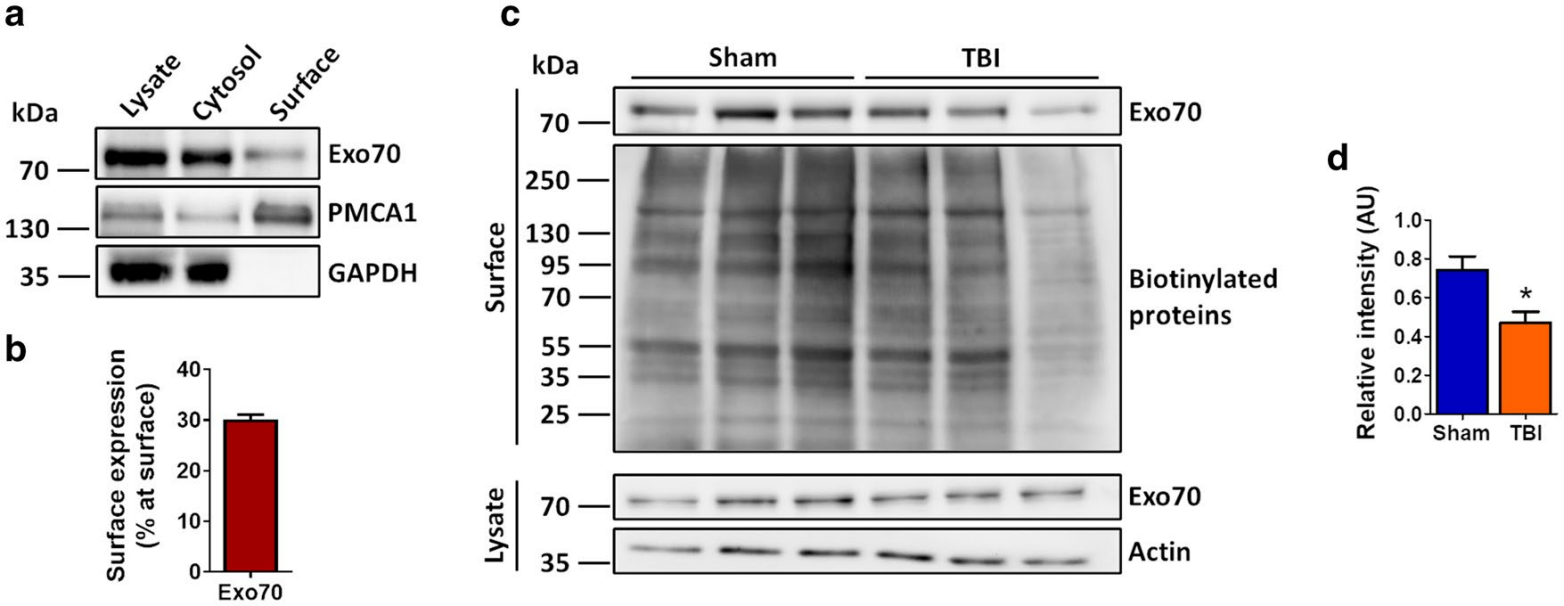
Exo70 proximity to the plasma membrane decreases after mTBI induction. **a** Brains from two-month-old mice were cut in 300 µm thick slices. Biotinylation was carried out and the hippocampus dissected. Hippocampal slices were homogenized in a modified RIPA buffer (see methods). 500 µg of protein samples were incubated with NeutrAvidin overnight at 4 °C and beads were obtained by brief centrifugation. 200 µg (40%) of total protein samples were loaded alongside cytosolic (unbound) and eluted samples from NeutrAvidin precipitation assays. Samples were resolved in 10% SDS-PAGE and transferred to PVDF membranes. Membranes were incubated with the antibodies indicated in the figure. Membranes were stripped and tested again with the indicated antibodies. **b** Exo70 proximity to the plasma membrane was described as follows: Combined cytosolic and surface Exo70 band intensities were considered as 100% Then Exo70 proximity was calculated using the band intensity combination. The graph shows that nearly 30% of the Exo70 total pool is proximal to the plasma membrane. Mean ± SEM is shown, n = 3 hippocampi from different mice. **c** Two-month-old male mice were subjected to mTBI and hippocampal slices were prepared and biotinylated. 500 µg of protein samples were incubated with NeutrAvidin overnight at 4 °C and beads were obtained by brief centrifugation. Lysates were analyzed using 30 µg of proteins. **d** Exo70 densitometric analysis. Protein lysates were used to normalize band intensities. 4 Hippocampal slices from each mouse were used. Values represent means ± SEM, n = 3 mice per experimental group. Statistical differences were determined by an unpaired t-test comparing Sham and mTBI. *  < 0.05

Exo70 is mainly found in the microsomal fraction as shown above. Microsome fraction contains endoplasmic reticulum, Golgi apparatus, synaptic vesicles, and plasma membrane [48]. As Exo70 is predominantly located at the plasma membrane [17, 18, 25], we wondered if Exo70 membrane localization is altered following traumatic brain injury. To test this, we used an ex vivo approach by which plasma membrane proteins are isolated using biotin. Brain from Sham and mTBI mice were cut to obtain slices and biotinylation was carried out (see methods). After biotinylation, hippocampal slices were dissected and homogenized, and biotinylated proteins were captured using NeutrAvidin. Using this approach, we were able to isolate the majority of the membrane fraction as shown by PMCA1, while cytosolic GAPDH showed absence in this fraction (Fig. 5a) indicating that the protocol worked as expected. Exo70 membrane localization showed to be nearly 30% of total Exo70 (Fig. 5b). Interestingly, after mTBI induction, Exo70 surface localization was reduced by 36% (Fig. 5c, d) suggesting that Exo70 redistribution towards PSD observed in fractionation experiments comes from the plasma membrane.

### Exocyst assembly following mild traumatic brain injury

**Fig. 6:**
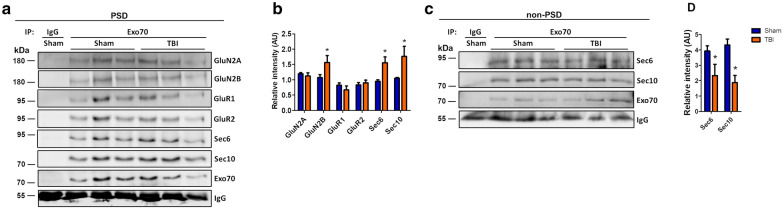
mTBI increases exocyst complex assembly and interaction between Exo70 and GluN2B in the PSD fraction. Two-month-old male mice were subjected to mTBI and hippocampal PSD/nonPSD fractions were obtained. 50 and 300 µg of PSD **(a)** and nonPSD **(c)** protein samples, respectively, were immunoprecipitated using a specific Exo70 polyclonal antibody. Beads were recovered by brief centrifugation. Samples were resolved in 6% SDS-PAGE and transferred to PVDF membranes. Signal intensity was normalized with Exo70 band intensity. Both hippocampi of each mouse were used. Membranes were stripped and tested again with the indicated antibodies. **b**, **d** GluN2A, GluN2B, GluR1, GluR2, Sec6, and Sec10 densitometric analysis from PSD and nonPSD immunoprecipitations. The analysis shows the assembly level of the exocyst complex and Exo70-GluN2B increased interaction after mTBI. Values represent means ± SEM, n = 3 mice per experimental group. Statistical differences were determined by an unpaired t-test comparing Sham and mTBI. *p < 0.05

Exo70 has been proposed as the nucleating factor of the exocyst complex establishing the site where the complex should be directed [21, 49–51]. Also, the level of assembly of the complex through Exo70 denotes its activity on exocytosis [15, 16, 52]. Therefore, we sought to determine if the assembly of the exocyst complex is altered on mTBI mice. In order to do that, hippocampal PSD and nonPSD fractions from Sham and mTBI mice were prepared as above and Exo70 was immunoprecipitated using a specific polyclonal antibody. Experiments were analyzed using Sec6 and Sec10 antibodies (Fig. 6a, c). In PSD, Exo70 interaction with Sec6 and Sec10 was increased indicating that the complex’s assembly is increased following mTBI (Fig. 6b). On the other hand, Exo70 interaction with Sec6 and Sec10 was reduced in nonPSD fraction (Fig. 6d). These results suggest that the complex assembly occurs in hippocampal PSD after mTBI induction.

The exocyst has been related to AMPA and NMDA receptors trafficking and delivery into the synapse [11, 12] by being in the same protein complex, thus modulating the availability of these receptors on PSD. We asked if mTBI could change the level of interaction between Exo70 and both types of ionotropic glutamate receptors in hippocampal PSD and nonPSD fractions. To address this, hippocampal PSD and nonPSD fractions were prepared and Exo70 was again immunoprecipitated. In these experiments, GluR1/GluR2 from the AMPA receptors and GluN2A/GluN2B from the NMDA receptors were assessed by western blot (Fig. 6a). The analysis showed that Exo70 doesn’t change its interaction with both GluR1 and GluR2 AMPAR subunits in PSD when mTBI was induced (Fig. 6b). When comes to the NMDA receptors, Exo70 interaction is increased only with the GluN2B subunit and not with GluN2A (Fig. 6b). We couldn’t detect any interaction between Exo70 and AMPA/NMDA subunits in nonPSD fraction (Additional file 1: Fig. S1). This suggests that the assembly of the exocyst complex favors the interaction with GluN2B in the hippocampus upon mTBI induction.

## Discussion

The exocyst complex basic function is the tethering of secretory vesicles during the process of membrane addition for polarized outgrowth [53], and its protein components have been involved in specialized membrane processes in neurons. As it so, the complex acts as a unit giving the molecular machinery to carry on trafficking and exocytosis related processes [45]. Here, we report a specific task that the exocyst is doing on acute neuropathology like traumatic brain injury. We found that Exo70 is redistributed in the hippocampus of mice subjected to mTBI and that the exocyst function might be altered on hippocampal synapses. In the present work, we used a modified Maryland’s weight drop model that was fitted to mice anatomy. Brain injury was developed with a closed head frontal impact device using a repeated strike paradigm (see methods). The usage of this weight drop model grants the ability to induce repeated strikes which is associated with accumulative damage [30]. The first injury is associated with the release of excitotoxic neurotransmitters, neurometabolic crisis, inflammation and axonal dysfunction [54]. A second TBI, that occurs before the resolution of the pathophysiological changes induced by the first TBI, adds cumulative damage to the brain and prolongs the recovery from the second injury [30, 55, 56]. Our protocol lacks any craniotomy procedures and consists of 5 days strike with a 2-day-interval which consequently adds damage to an already damaged system. In the end, the protocol resembles severe brain damage without any aggressive surgical procedures [44].

Exo70 expression has been reported on many mono and multicellular organisms including mammals and it is considered as a ubiquitous protein expressed in most cell types including brain cells [45]. Several reports have indicated that Exo70 is present in brain tissue and specifically in neurons [11, 12, 20, 21, 57–59], but none have studied its expression patterning in the cortex and hippocampus. We showed that Exo70 is expressed both in the cortex and hippocampus by western blot (Fig. 1c). By immunochemistry, Exo70 appears to be expressed in most of the cortical layers (Fig. 1a). In the hippocampus, Exo70 is expressed mainly in *stratum pyramidale* which contains most of the neuronal soma present in CA1-CA3 hippocampal zones and thus we believe that Exo70 is predominantly expressed in neurons, although some Exo70-positive cells were detected outside of *stratum pyramidale* (Fig. 1b). We are aware that it’s necessary to triple-label brain tissue to have a more comprehensive expression of Exo70 between brain cells, yet this is beyond the scope of this study. This study was focused on mTBI and its effect on Exo70 expression/function. First, we searched for Exo70 expression changes in the forebrain, cortex, and hippocampus upon mTBI induction and we found no disturbances (Fig. 2).

The lack of expression disturbances doesn’t necessarily mean that Exo70 has no changes in mTBI. Exo70′s function is strictly related to its subcellular localization and changes in this localization have shown to be important to developing Exo70 malfunction [14, 20, 22, 23, 60]. As we found no expression changes, we wondered if Exo70 subcellular distribution is altered after mTBI. We used a well-established protocol to isolate subcellular organelles and fractions such as microsome and synaptosomes. Exo70 showed to be mainly located at microsomes followed by synaptic localization which has also been seen in the rat brain [21], supporting the idea that Exo70 function is related to these subcellular compartments. We observed that in the forebrain, Exo70 increased its localization in microsome and synaptosome fractions upon TBI induction (Fig. 3). These fractions are mainly composed of cellular membranes related to intracellular and synaptic trafficking, such as Golgi to membrane trafficking, exocytosis, and endosome recycling of cargoes directed to synapses [48]. Particularly Exo70 functions have been described in these processes where cargoes are transported with the exocyst complex [13], but whether TBI shows alterations in dendritic trafficking related to these fractions it’s not totally understood. One possibility is that TBI induces Exo70 accumulation into these membrane fractions to change trafficking through Exo70 and the exocyst complex as a result of the pathophysiology development, and the other possibility is that Exo70 could be part of a compensatory mechanism to counteract damage development. The forebrain contains many zones where different biological processes are carried out, thus, our results can’t determine which possibility is the correct one for forebrain fractionation.

TBI causes neuronal damage principally in the cortex [43, 61, 62] but also the damage reaches the hippocampus eventually [43, 44, 63–66]. Consequently, we sought to determine whether Exo70 distribution gets differentially affected between cortex and hippocampus. It is worth noting that differences are observed in Exo70 subcellular distribution between the forebrain and hippocampi. In the forebrain, Exo70 was barely seen in the cytosolic fraction, whereas more presence was observed in the hippocampal cytosolic fraction. All of this despite Exo70 being highly distributed in microsomes in both samples. It is likely that forebrain, as a multizone tissue, englobes many Exo70 subcellular distribution patterns, while Exo70 hippocampal distribution is essentially granted to an isolated zone. Regarding TBI, we determined that Exo70 is redistributed into synapse only in the hippocampus and that this pool might come from microsomal fraction (Fig. 4). Diminishing Exo70 localization in microsomes would allow cellular systems to provide the pool of Exo70 necessary to the redistribution into the postsynaptic density in the synaptic compartment. This redistribution found in the hippocampus was not seen in the cortex, therefore there might be some tissue-specific alterations related to Exo70 in the TBI context. The fact that Exo70 distribution doesn’t change in the cortex raises the question: Is Exo70 important in the development of TBI pathology? We suggest that Exo70 localization is altered to modify its function, probably providing the site where the exocyst must be redirected to play a role in the development of the pathology that has not been discovered yet. Forebrain fractionation showed that Exo70 localization in microsomes is increased, contrary to the reduction observed in hippocampal microsomes of mice subjected to our repeated mTBI protocol. This difference relies on the forebrain (without the cerebellum) being composed of many zones, which number in its entirety could obscure an event occurring in specific tissues such as the hippocampus.

Microsome fraction is a technical fraction obtained only by subcellular fractionation and thus is not a “real” cellular compartment. As tissue homogenization is carried out with an isotonic buffer, membranous organelles remain fairly untouched and thus several organelles are isolated together. Microsome fraction contains endoplasmic reticulum, Golgi apparatus, synaptic vesicles, and plasma membrane [48]. In our TBI model, we found that Exo70 localization is reduced in microsomal fraction (Fig. 4) and that Exo70 is mainly located on this fraction on Sham animals. As Exo70 is predominantly located at the plasma membrane [17, 18, 25], we hypothesized that the reduction of Exo70 in microsomes is due to a decrease in plasma membrane localization after mTBI. In this case, we used a biotinylation protocol that can isolate plasma membrane proteins. This protocol biotinylated extracellular portions of those proteins and therefore intracellular proteins should not be biotinylated (Fig. 5a) [67, 68]. It is important to note that Exo70 is an intracellular protein. Nevertheless, this biotinylation protocol has been used to evaluate the membrane localization of intracellular proteins such as Fyn kinase and Tau [69] due to the ability of these proteins to interact with surface biotinylated partners. Indeed, Fyn is an intracellular kinase that interacts and phosphorylates NMDAR [31, 70, 71]. Additionally, exocyst subunits and other “cytosolic” proteins have also been detected in surface membrane fraction in cortical tissue [72]. Exo70, as an exocytic protein, is thought to interact with several membrane proteins and there is some evidence of it [11, 12, 14, 24, 73] and thus we consider this protocol suitable for assessing Exo70 membrane localization. We showed that Exo70 membrane localization is reduced on the hippocampus when mTBI was induced (Fig. 5d) suggesting that the pool of Exo70 that is redistributed from the microsome into synapses comes from the plasma membrane. Altogether, our biochemical experiments showed that Exo70 function might be altered in the mTBI context.

The mammalian exocyst is a multimeric complex that has been shown to have two subcomplexes. Subcomplex 1 (SC1) comprises Sec3, Sec5, Sec6 and Sec8; subcomplex 2 (SC2) comprises Sec10, Sec15, Exo70 and Exo84 [50]. When SC1 and SC2 assembly is induced, the complex is activated. In this work, we analyzed Sec6 and Sec10. Both Exo70 and Sec10 lays on SC2 while Sec6 is present in SC1, therefore our western blot analysis to Sec6 and Sec10 is suitable to evaluate the assembly of the exocyst. This approach has been used before on biochemical experiments with a similar aiming on exocyst assembly and activity [15, 16, 52, 74]. The exocyst is a complex widely distributed intracellularly [12, 21], so immunoprecipitating Exo70 in hippocampal protein extracts would add several difficulties in assessing the particular assembly on synapses. Hence, we decided to narrow down the possibilities and isolated synaptosomes to address this issue. Hippocampal synaptosomes were Triton-treated and PSD/nonPSD fractions were obtained before Exo70 immunoprecipitation. Our results showed that upon mTBI induction, exocyst assembly increased only on PSD and decreased in nonPSD fraction (Fig. 6). This suggests that the Exo70 redistribution towards PSD provides a site where the exocyst complex can increase its assembly and probably its activity, triggering the redirection of nonPSD ensembled complex into PSD. The activity of the complex has been shown to depends on its assembly and therefore we suggest that the activity of the complex is increased after mTBI on hippocampal PSD. Due to this increased activity specifically in PSD, one could speculate that the exocyst could reinforce the stabilization of the PSD structure by providing with cargoes that are required to stabilize synapses from the postsynaptic compartment.

One possibility is that the exocyst could provide the machinery to modulate the availability of glutamate receptors at PSD. The exocyst has been related to traffic and delivery of AMPA and NMDA receptors into the synapse [11–13]. These studies suggested that Sec6, Sec8, and Exo70 might be in the same protein complex with AMPAR subunits such as GluR1 and GluR2 [11]. Additionally, NMDAR subunits GluN1/GluN2B also has been detected in the same complex with Sec6, Sec8, and Exo70 [12]. Glutamate receptors have been extensively studied in several traumatic brain injury models with proteomic, electrophysiological, and behavioral approaches [30, 31, 54]. Basal synaptic transmission and plasticity are decreased upon TBI induction [44, 75] which is accompanied by a reduction in glutamate binding to NMDA receptor [76] and ultimately internalization of glutamate receptors. GluR2 is internalized after TBI [37]. GluN2B availability at synapses is reduced dramatically upon TBI induction [41]. Both events trigger downstream signaling that ultimately induces cell death [31]. Thus, given the fact that Exo70 and the exocyst are part of AMPAR/NMDAR trafficking and delivery machinery [13], one could speculate that the induction of exocyst assembly on PSD is likely a compensatory event by which glutamate receptors are maintained in the synapse to diminish TBI detrimental signaling. To test this, we immunoprecipitated Exo70, and membranes were probed with GluR1, GluR2, GluN2A, and GluN2B antibodies (Fig. 6). The results showed that only GluN2B increased its interaction with Exo70 on PSD. The same interactions were tested on nonPSD fraction and no signal was detected, suggesting that the exocyst function through Exo70 towards PSD in order to maintain synapse stability. This has been proven at least in hippocampal neuron cultures [21]. We suggest that exocyst assembly favors Exo70-GluN2B interaction to develop a compensatory mechanism by which GluN2B gets stabilized in the PSD rather than an evoked increase of this receptor at the PSD. This, in turn, prevents the development of the pathology.

## Conclusions

Summarizing, the present findings position Exo70 in the group of proteins that could modulate GluN2B synaptic availability. As Exo70 has been proven to be redistributed from microsomes towards PSD, one would anticipate that there would be a special function at these specific compartments. Microsomal fraction contains the plasma membrane, which is the main Exo70 localization and thus the pool of Exo70 used to be redistributed comes from the plasma membrane. One scenery gives Exo70 the ability to counteract GluN2B redistribution away from the synapse by inducing synaptic assembly of the exocyst complex and thus we suggest that Exo70 redistribution into the synapse is part of a compensatory mechanism that prevents the development of TBI pathology. Future work would address this statement from a physiological point-of-view. Finally, we are the first to describe *in* vivo brain redistribution of Exo70 in acute neuropathology like TBI.

## Material and methods

### Antibodies

Primary antibodies: rabbit Exo70 1:1000 (Cat 12014-1-AP, Proteintech, USA), rabbit GAPDH 1:1000 (Cat sc-48166, Santa Cruz, USA), mouse Tubulin 1:1000 (Cat NB500-333, NovusBio, USA), mouse PSD-95 1:500 (Cat sc-32290, Santa Cruz, USA) mouse synaptophysin 1:500 (Cat sc-55507, Santa Cruz, USA), rabbit PDI 1:1000 (Cat sc-20132, Santa Cruz, USA), mouse β-Actin 1:1000 (Cat A3853, Sigma, USA), mouse PMCA1 1:1000 (Cat sc-398413, Santa Cruz, USA), mouse GluR1 1:1000 (Cat N355/1, NeuroMabs, USA), mouse GluR2 1:500 (Cat sc-517265, Santa Cruz, USA), mouse GluN2A 1:1000 (Cat N327/95, NeuroMabs, USA), rabbit GluN2B 1:1000 (Cat A6474, Invitrogen, USA), mouse Sec6 1:500 (Cat sc-393230, Santa Cruz, USA) and mouse Sec10 1:500 (Cat sc-514802, Santa Cruz, USA). All secondary antibodies were obtained from Jackson ImmunoResearch, Baltimore, USA.

### Animals

Two months old C57BL/6 J male mice were used in this study. Animals were housed up to 4 mice per cage with a 12:12 h light/dark cycle (light on at 8:00 am) and provided food and water ad libitum. Animals were obtained from CEBIM-UC (Center for Innovation in Biomedical Experimental Models from the Pontificia Universidad Católica de Chile). Animals were handled according to the National Institutes of Health guidelines (NIH Publications No. 8023, revised 1978, Baltimore, MD). All experimental procedures were approved by the Bioethical and Biosafety Committee of the Faculty of Biological Sciences of the Pontificia Universidad Católica de Chile (181,009,010).

### Immunohistochemistry

Perfusion, fixation, and free-floating immunofluorescence were performed as described previously [34]. Briefly, 30 µm thick brain slices were permeabilized with 0.2% (v/v) Triton X-100 in PBS (PBS-T) for 30 min. Then, a 30 min incubation was carried out with 0.15 M Glycine and 10 mg/ml NaBH_4_ to decrease background auto-fluorescence. Slices were washed with PBS-T three times and blocked with 3% BSA for 1 h at room temperature. Slices were incubated overnight at 4 °C with Exo70 primary antibody diluted in PBS-T containing 3% BSA. After three PBS-T washes, slices were incubated with a secondary antibody in PBS-T containing 3% BSA for 2 h at room temperature. Slices were then washed three times with PBS and distilled water. Hoechst was used to stain the nucleus. Finally, slices were mounted on gelatin-coated slides using Dako Fluorescence Mounting Medium (Dako, CA, USA) and images were captured in an Olympus BX51 microscope equipped with a Micro-publisher 3.3 RTV camera (QImaging, Surrey, BC, Canada) using Q-imaging software.

### Repeated mild traumatic brain injury

We used a modified Maryland’s weight drop model [43]. For this purpose, we adapted the impact device to fit mice anatomy. Animals were randomly assigned to receive either sham or TBI. Mice were anesthetized with isoflurane using the open-drop method [77]. Animals were then gently restrained in position onto the impact device using an elastic belt placed across the dorsal thorax leaving the head free. Mice were subjected to 5 sessions of 3 blasts each with a 2-day interval in a frontal weight impact device. After each session, mice were monitored carefully until the anesthesia effect was finalized and returned to their home cage. Then, animals were kept in their home cage for 7 days before analysis. Sham animals were subjected to all procedures except injury induction. Animals were sacrificed by decapitation and brains were rapidly dissected in cold artificial cerebrospinal fluid (ACSF, in mM: 124 NaCl, 2.6 NaHCO_3_, 10 D-glucose, 2.69 KCl, 1.25 KH_2_PO_4_, 2.5 CaCl_2_, 1.3 MgSO_4,_ and 2.60 NaHPO_4_).

### Western blot analysis

Brains, cortex, and hippocampi of sham and TBI mice were dissected on ice and immediately processed. Tissues were homogenized in RIPA buffer (10 mM Tris–HCl, Triton X-100 0.5%, 1% NP-40, 5 mM EDTA, 1% sodium deoxycholate, and 1% SDS) supplemented with protease and phosphatase inhibitor mixture (Protease: Amresco, VWR Life Science; Phosphatase: 25 mM NaF, 100 mM Na_3_VO_4_ and 30 µM Na_4_P_2_O_7_) using a Potter homogenizer and then passing through a tuberculin syringe. Samples were centrifuged at 14,000 rpm at 4 °C for 10 min. Protein concentration was determined using a BCA protein assay kit (Pierce, ThermoFisher Scientific, USA). Samples were resolved by SDS-PAGE and transferred to PVDF membranes. Western blot was performed as previously described [21] with overnight incubation of primary antibodies at 4 °C. Blots were developed using a chemiluminescence detection kit (West Pico, ThermoFisher, USA). Images were obtained with a G:BOX Chemi XT4 Gel imaging system (Syngene). Membranes were stripped for 30 min at room temperature using a harsh stripping buffer (6 M GnHCl, 0.2% NP-40, 100 mM β-mercaptoethanol, 20 mM Tris–HCl, pH 7.5) and washed thoroughly 4 times with PBS containing 0.1% Tween-20. After stripping, membranes were tested again with different antibodies where needed.

### Subcellular fractionation

For subcellular fractionation, the sucrose gradient was performed as described previously [21, 47]. Briefly, forebrains were rapidly dissected on cold ACSF. The brains were homogenized in buffer A containing 5 mM Hepes, pH 7.4; 320 mM sucrose supplemented with a protease and phosphatase inhibitor mixture. Homogenization was done using a potter S by delivering 10 up and down strokes. Cell debris were removed by centrifugation for 10 min at 1,000 g (P1) and the supernatant (S1) was centrifuged for 20 min at 20,000 g obtaining S2 (cytosol and microsomes) and P2 fractions. In order to isolate microsomes, the S2 fraction was centrifuged for 2 h at 100,000×g. P2 fraction was loaded on top of a 0.32/0.85/1.0/1.2 M sucrose gradient and centrifuged for 2 h at 100,000×g and synaptosomes were obtained from 1.0/1.2 M interface. Synaptosomes were then resuspended in Triton buffer (20 mM HEPES, 100 mM NaCl, 0.5% Triton X-100, pH 7.2) and rotated for 20 min before centrifugation at 20,000×g for 20 min to obtain PSD and nonPSD fractions. PSD pellet was resuspended with PBS unless otherwise mentioned. All manipulations were carried out on the ice or at 4 °C. Samples were stored at -80 °C until use. Proteins were quantified and analyzed by Western blot.

### Co-immunoprecipitation

Co-immunoprecipitation experiments were carried out as follows. Mice brains were removed and both hippocampi were dissected. PSD and nonPSD fractions were prepared as described above with minor modifications. PSD fractions were resuspended in RIPA buffer (25 mM Tris–Cl, pH 7.6, 150 mM NaCl, 1% NP-40, and 0,1% SDS) and rotated for 20 min for immunoprecipitation. Samples were quantified using the BCA Protein assay kit (Pierce, ThermoFisher Scientific, USA). 50 µg of PSD and 300 µg of nonPSD protein samples were incubated with 1 µg of Exo70 antibody overnight at 4 °C followed by incubation with Protein A/G agarose beads (30 µl; Santa Cruz Biotechnology, USA) for 1 h at 4 °C. Normal rabbit IgG was used as a control for immunoprecipitation. Then, beads were recovered by brief centrifugation and washed three times with PBS. Proteins were eluted with a 2 × protein loading buffer.

### Cell surface biotinylation

Hippocampal slices were prepared as described previously [34]. Briefly, brains were quickly removed and transverse slices (300 µm) were cut under cold ACSF using a vibratome (BSK microslicer DTK-1500E, Ted Pella, Redding, CA, USA) and incubated in a resting chamber for 1 h with a 95% O_2_/5% CO_2_ saturation. Biotinylation was carried out as described previously with minor modifications [72]. Briefly, brain slices were transferred to an incubation chamber at 4 °C supplemented with O_2_ and CO_2_. Then, slices were incubated with EZ-Link Sulfo-NHS-Biotin (1 mg/ml in ACSF, ThermoScientific) for 45 min to biotinylated surface proteins. Excess biotin was removed by washing the slices three times (5 min) with a quenching solution (Glycine 100 mM) and then washing three times with cold ACSF. Hippocampal slices were dissected under a binocular microscope (Amscope, Irvine, CA, USA). Slices were homogenized in modified RIPA buffer (150 mM NaCl, 20 mM HEPES, 1% Triton X-100, 0.5% SDS, and 2 mM EDTA, pH 7.4) supplemented with a mixture of protease and phosphatase inhibitors. Sample homogenates (total fraction) were collected and cell debris was removed by centrifugation for 10 min at 12,000 g. Next, supernatant proteins were quantified using a BCA protein assay kit (Pierce, ThermoFisher Scientific, USA). 500 µg of protein were incubated overnight at 4 °C with Neutravidin Agarose beads (50 µl; Pierce, ThermoFisher Scientific, USA). Beads were recovered by brief centrifugation and washed three times with ice-cold PBS. Proteins were eluted with a 2 × protein loading buffer.

### Image and statistical analysis

Densitometry analysis was carried out with ImageJ (NIH) software. Data were normalized with specific loading control indicated in each figure legend. All data are expressed as mean ± SEM. The difference between groups was determined by an unpaired t-test to establish significant differences. A p < 0.05 value was considered significant. The statistical test was completed using Graphpad 5 software. Statistical power was calculated to discard the null hypothesis using PASS 2019 Power Analysis and Sample Size Software (NCSS, LLC. Kaysville, Utah, USA). Each data shown with significant differences was running into power analysis and the results are as follows: Fig. 3, microsome and synaptosome 100% of power; Fig. 4, microsome 99.53% and PSD 100% of power; Fig. 5 biotinylation 99.48% of the power. Finally, Fig. 6 power analysis results for PSD: GluN2B 97.74%, Sec6 95.72%, and Sec10 85.44%. For nonPSD: Sec6 91.33% and Sec10 96.05%.

## Supplementary Information

**Additional file1**: **Figure S1**. Null detection of ionotropic glutamate receptors in nonPSD fraction. Two-month-old male mice were subjected to mTBI and hippocampal nonPSD fraction was obtained. Membranes correspond to the experiments shown in figure 6C. We couldn’t detect any signal from the glutamate receptors analyzed (TIF 462 KB)

## Data Availability

All data supporting the conclusions of this article are included within the manuscript and are available upon reasonable request to the corresponding author.

## Abbreviations

SNARE: Soluble NSF attachment protein receptor
Glut4: Glucose transporter type 4
TBI: Traumatic brain injury
mTBI: Mild traumatic brain injury
CA1: Cornu Ammonis 1
CA3: Cornu Ammonis 3
NMDAR: N-methyl-D-aspartate receptor
AMPAR: α-Amino-3-hydroxy-5-methyl-4-isoxazolepropionic acid receptor
PSD95: Postsynaptic Density 95
PDI: Protein Disulfide Isomerase
PMCA1: Plasma Membrane Calcium ATPase 1
GAPDH: Glyceraldehyde 3-phosphate dehydrogenase
PSD: Postsynaptic density
nonPSD: Non-postsynaptic density
SC1: Subcomplex 1
SC2: Subcomplex 2
