# Supplementary figures and images for: Exo70 intracellular redistribution after repeated mild traumatic brain injury
Source: Biol Res. 2021 Feb 16;54:5. doi: 10.1186/s40659-021-00329-3 (PMC7885507; doi:10.1186/s40659-021-00329-3)

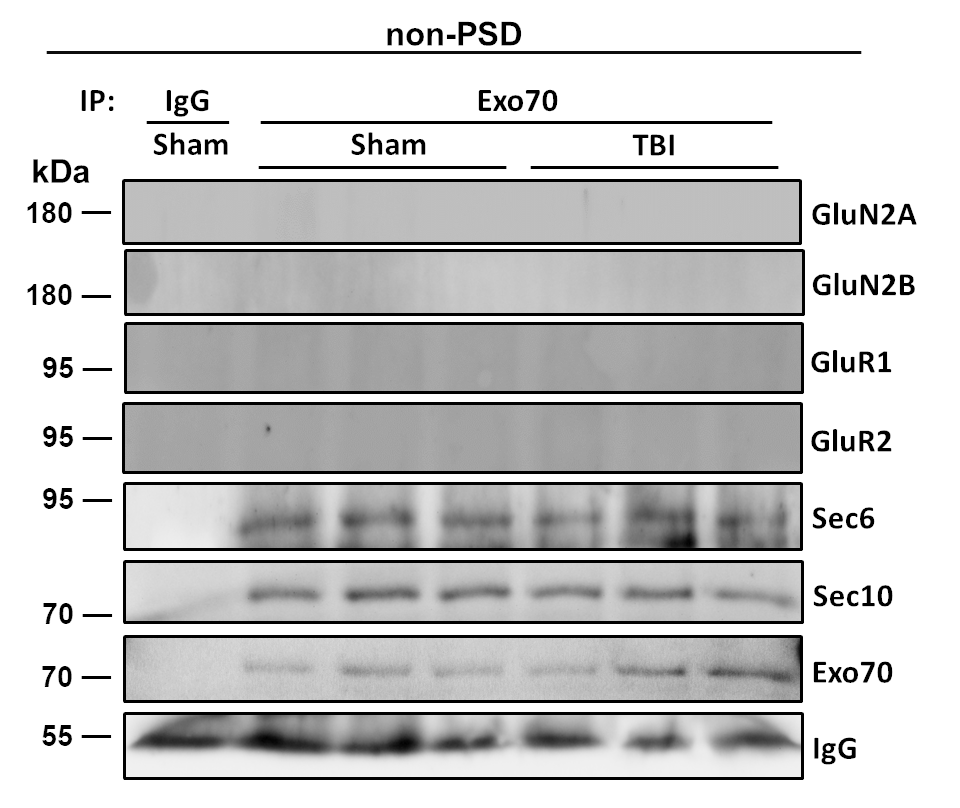

Supplement: Supplementary file 1 — Additional file1: Figure S1. Null detection of ionotropic glutamate receptors in nonPSD fraction. Two-month-old male mice were subjected to mTBI and hippocampal nonPSD fraction was obtained. Membranes correspond to the experiments shown in figure 6C. We couldn’t detect any signal from the glutamate receptors analyzed (TIF 462 KB) [file 40659_2021_329_MOESM1_ESM.tif]
